# ISWI and CHD chromatin remodelers bind to promoters but act in gene bodies

**DOI:** 10.1186/1756-8935-6-S1-O29

**Published:** 2013-03-18

**Authors:** Gabriel E Zentner, Toshio Tsukiyama, Steven Henikoff

**Affiliations:** 1 Basic Sciences Division, Fred Hutchinson Cancer Research Center, Seattle, WA 98109, USA; 2 Howard Hughes Medical Institute, Fred Hutchinson Cancer Research Center, Seattle, WA 98109, USA

## Background

ATP-dependent nucleosome remodelers influence genetic processes by altering nucleosome occupancy, positioning, and composition. *In vitro*, yeast imitation switch (ISWI) and chromodomain helicase DNA-binding (CHD) remodelers bind ~30-85 bp of extranucleosomal DNA [1-3]. However, *in vivo*, ISWI and CHD remodelers act within gene bodies [4], which contain regularly spaced nucleosomes separated by less than 20 bp of linker DNA [5]. How, then, do ISWI and CHD remodelers act within regions containing insufficient linker DNA for their association with chromatin?

## Methods

To resolve this apparent paradox, we have mapped the genomic distributions of the yeast Isw1, Isw2, and Chd1 remodelers at base-pair resolution using immunoprecipitation of MNase-digested native chromatin and high-throughput sequencing (N-ChIP-seq).

## Results

We find that ISWI and CHD remodelers are most highly enriched at nucleosome-depleted regions (NDRs), where they bind to regions of extended linker DNA adjacent to particular transcription factors. Surprisingly, remodeler-NDR association is independent of catalytic activity, suggesting that remodeler binding to NDRs is not catalytically relevant but instead poises remodelers for action within gene bodies. Remodeler occupancy is correlated with nucleosome turnover, suggesting that remodelers act within regions of transient nucleosome depletion following transcriptional elongation.

## Conclusions

We suggest that transcription factors recruit ISWI and CHD remodelers to their binding sites within NDRs, where there is ample linker DNA to facilitate their association with chromatin. Transcriptional elongation then evicts or disrupts nucleosomes, creating regions of extended linker DNA within gene bodies. NDR-bound ISWI and CHD remodelers would thus be poised to follow in the wake of RNA polymerase II, efficiently repositioning nucleosomes within regions of extended linker DNA. This model reconciles the seemingly incompatible *in vitro* extranucleosomal DNA requirements of ISWI and CHD remodelers with the short linkers found *in vivo*.
